# Force-induced remodelling of proteins and their complexes

**DOI:** 10.1016/j.sbi.2015.02.001

**Published:** 2015-02-21

**Authors:** Yun Chen, Sheena E Radford, David J Brockwell

**Affiliations:** 1 Astbury Centre for Structural Molecular Biology, University of Leeds, Leeds LS2 9JT, UK; 2 School of Molecular and Cellular Biology, University of Leeds, Leeds LS2 9JT, UK

## Abstract

Force can drive conformational changes in proteins, as well as modulate their stability and the affinity of their complexes, allowing a mechanical input to be converted into a biochemical output. These properties have been utilised by nature and force is now recognised to be widely used at the cellular level. The effects of force on the biophysical properties of biological systems can be large and varied. As these effects are only apparent in the presence of force, studies on the same proteins using traditional ensemble biophysical methods can yield apparently conflicting results. Where appropriate, therefore, force measurements should be integrated with other experimental approaches to understand the physiological context of the system under study.

## Introduction

The first mechanical unfolding experiment was carried out almost 20 years ago and since this time great strides have been made in understanding the relationship of mechanical stability to the structure of proteins/protein complexes and their thermodynamic or kinetic stability (see [1] and [2]). This knowledge is important because the mechanical perturbation of bio-molecular conformation affects the stability of bio-molecules, their affinity for ligands and binding partners and even their catalytic efficiency. As these parameters control many of the basic processes that occur in cells, force has been implicated in processes as diverse as catalysis, signal transduction, protein degradation and differentiation. Importantly, even the relatively small forces that are encountered *in vivo* can result in large changes to structure and/or affinity. Consequently, the application of force *in vivo* can alter biological activity in a manner that cannot be recapitulated in the absence of force *in vitro*. In the last few years our knowledge of the effects of force in biology, gained using single-molecule experimental and theoretical approaches, has allowed the effects of force to be delineated for relatively simple systems *in vitro*, as well as more complex systems found *in vivo*. In this review, we briefly describe the single molecule force techniques utilised to probe bio-molecular interactions and the underlying theories and models used to interpret the results. By reference to the recent literature, we then focus on the main topic of this review: progress in unravelling the roles of mechanical force in cellular systems.

## Measuring and analysing the effects of force on bio-molecules

**Figure 1:**
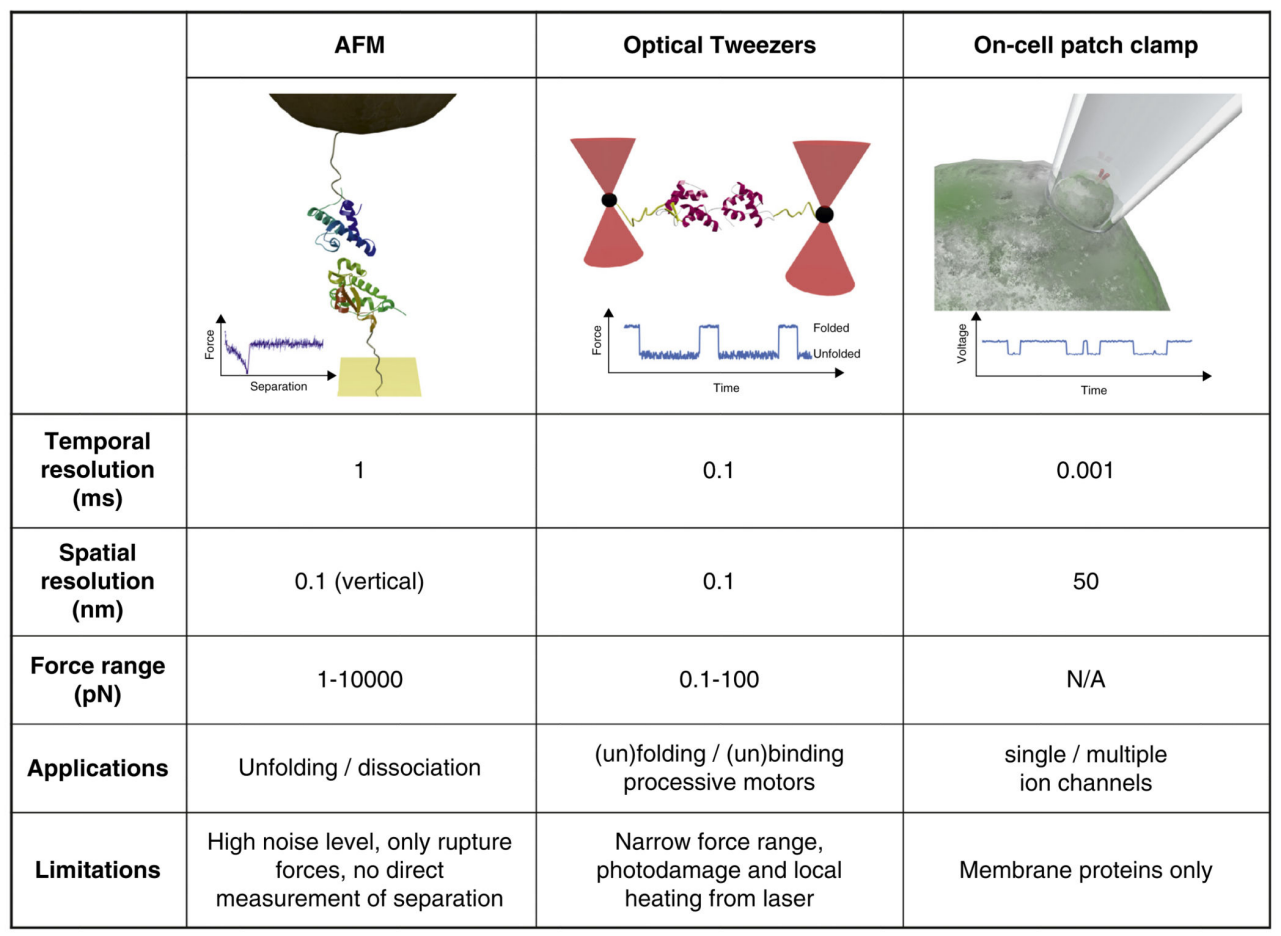
Experimental set-up and comparison of the key parameters, features and limitations of (left) atomic force microscopy (AFM), (middle) optical tweezers and (right) on-cell patch clamping.

To analyse and understand the effects of force at the molecular level it is necessary to manipulate single bio-molecules or perturb their environment. This review discusses three commonly used techniques that achieve this feat: atomic force microscopy (AFM), optical or magnetic tweezers and patch clamping. As summarised in Figure 1 each method has its own optimal force and distance resolutions and experimental limitations. The application of AFM to force measurements (sometimes called force spectroscopy or dynamic force spectroscopy, DFS) has gained widespread use in biology. It is able to measure single molecule stretching and rupture forces directly with subnanometer distance and picoNewton force resolution [3]. This technique is typically used to measure relatively high forces in biological terms (pN — nN) at relatively high extension rates (10–10000 nm s^−1^). However, both limitations have been addressed by the use of uncoated [4] and nano-engineered cantilevers [5^•^], pushing the lower force threshold to the subpicoNewton level. Tweezers use either light (laser tweezers/optical traps) or magnetic fields (magnetic tweezers) as the force transducer [6,7]. Laser tweezers have been used to study processive motors, such as myosin [8], kinesin [9] and ClpX [10,11], while magnetic tweezers are applied most often to torque-generating proteins such as those that interact with DNA [12]. Both techniques are now being applied to study mechanical unfolding, with optical trapping technology able to study multiple folding/unfolding cycles of a single protein over many seconds [13]. On-cell patch-clamp is an electrophysiological technique that is especially powerful for the study of single ion channels on intact cells. By attaching a micropipette to the cell membrane, the current created by a single ion channel can be recorded [14]. For force studies, patch clamp offers the opportunity to assess the role of the lipid environment on membrane proteins by changing membrane tension or modulating lipid identity. The utility of these techniques can also be enhanced by combining them with other methods. For example, the combination of force and fluorescence spectroscopy provides a powerful tool to gain information simultaneously on single molecule forces and conformational changes (including complex formation) induced by force [15,16].

**Figure 2:**
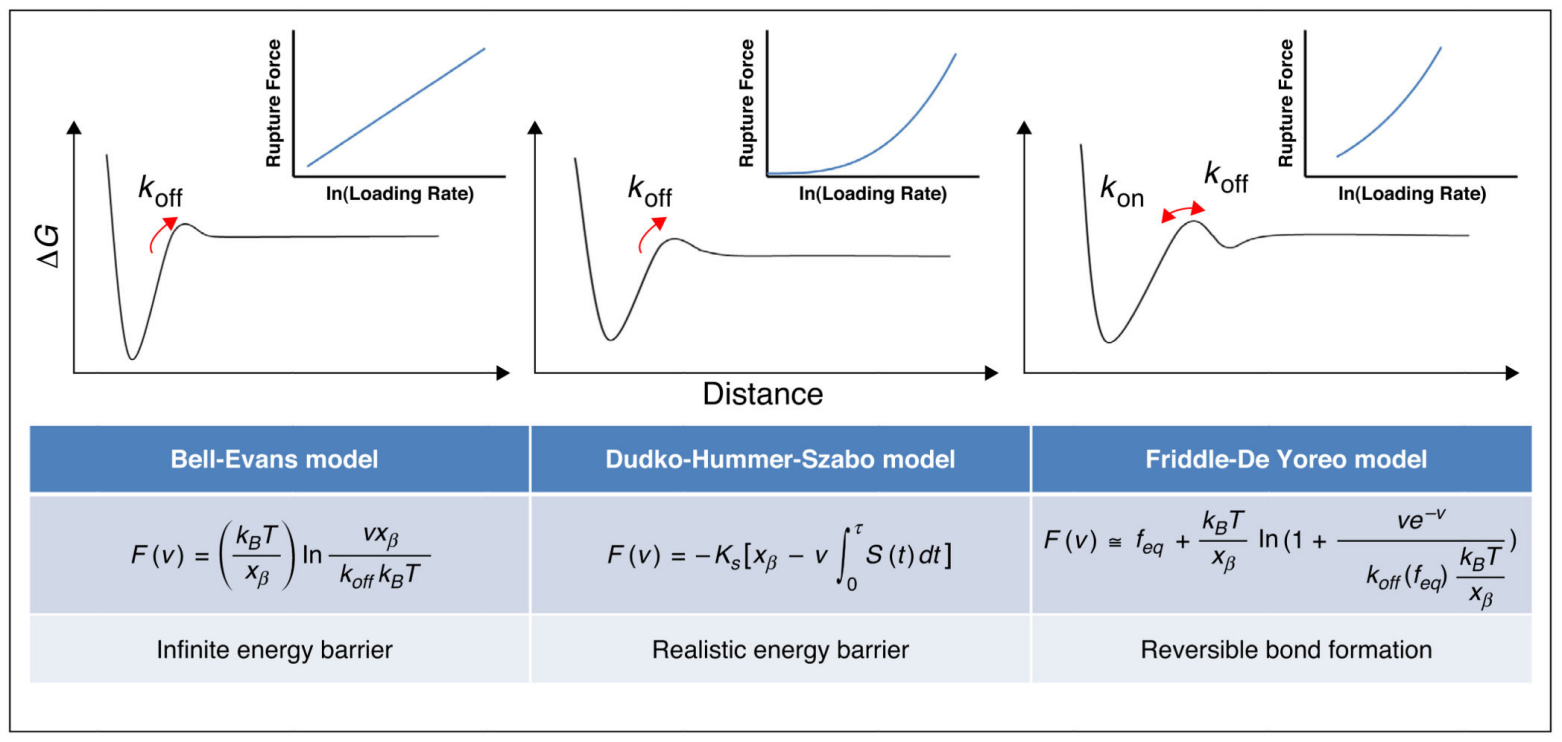
Models used to interpret DFS data. The assumed energy landscape and the resultant theoretical force versus loading rate relationship (insets) are shown above each model where *F*(*v*) is the most probable rupture force at a loading rate *v*, *k*_B_ is Boltzmann’s constant, *T* is temperature, *x*_β_ indicates the location of the energy barrier, and *k*_off_ is the off rate constant at zero force. In Dudko-Hummer-Szabo model (centre), *k*_s_ is the harmonic force constant scaled by *k*_B_*T* and *S*(*t*) is the rupture probability as a function of the time *t*. In Friddle-De Yoreo model (right), *f*_eq_ indicates the force at which the dissociation and association are in equilibrium, and k_off_(*f*_eq_) is the off rate constant at *f*_eq_.

For most interactions, force acts to decrease the stability of the folded or bound state of a protein or complex relative to the unfolded or dissociated state. The effect of force can be thought of as a mechanical lever that tilts the underlying energy landscape with a magnitude that increases with distance from the native (or bound) state. Thus, in addition to stabilising the unfolded state, force also reduces the free energy barrier to unfolding (or unbinding). As these experiments are usually carried out far-from-equilibrium at a variety of extension rates, it is necessary to calculate the parameters that describe the unperturbed energy landscape (*k*_off_ and *x*_β_, see Figure 2) so that the results from different experiments can be compared. In order to reveal kinetic parameters for the reactions of interest a dynamic force spectrum is measured by performing single molecule force spectroscopy at different loading rates [3]. A plot of most likely rupture force versus force loading rate can be fitted to different analytical models. In the Bell–Evans model (Figure 2 left), it is assumed that the energy barrier is so deep that its position does not change, but the height of the escape barrier is lowered by the applied force. The most probable rupture force is then proportional to the natural logarithm of the loading rate [17,18]. A modification of the Bell–Evans model by applying Kramer’s diffusion theory was later proposed (Figure 2 middle) to avoid this assumption [19,20]. Both models above ignore the possibility of reversible bond formation during the force spectroscopy, and have been challenged by the Friddle-De Yoreo model (Figure 2 right) [21]. In this recently developed model, it is assumed that at relatively low loading rates, there is another shallow barrier for rebinding; while at higher loading rates, this secondary barrier increases so that the probability of rebinding is reduced.

## How do proteins and complexes respond to force?

As can be inferred from the macroscopic world, the application of force usually decreases the lifetime of a non-covalent interaction (whether a single hydrogen bond or the multitude of interactions that stabilise the folded conformation of a protein or a bio-molecular complex). Bonds that show an exponential decrease in lifetime with increased force as described in the Bell model [17–18] and its more sophisticated variants [19–21] are known as ‘slip bonds’. In this case force acts as a denaturant by diminishing barriers to unfolding/unbinding to the extent that the protein unfolds/unbinds at some characteristic force or timescale due to thermal fluctuations. Application of even the relatively small forces that are usually applied to single complexes *in vivo* (<40 pN [22,23]) reduces the lifetimes of proteins and their complexes exponentially, allowing the dissociation of high affinity interactions to take place on a physiologically relevant timescale. This is exemplified by recent work from Tolar and colleagues [24^••^] who investigated the role of force in the early steps of the humoral immune response that culminates in the generation of high affinity antibodies. Antibody generation is initiated by the binding of B cell receptors (BCRs) to a specific antigen on the surface of antigen presenting cells (APCs). B cells were found to actively pull out finger-like protrusions from APCs before internalising both lipid and antigen. The authors were able to show that BCR:antigen complexes with higher affinities and, therefore, longer lifetimes under force (measured by DFS experiments using the AFM) showed a greater internalisation of antigen-containing membrane. As the lifetime of the force transduction network is longer-lived for cognate BCR:antigen interactions relative to low affinity non-cognate complexes, application of force allows selection of high affinity antibodies over a physiologically relevant timescale.

If the unfolding pathways in the presence and absence of force are identical (i.e. there is no force-induced remodelling) then the force-induced off rates, when extrapolated to zero applied force, should be identical to the intrinsic thermally-induced unfolding/unbinding rate, as found for the unbinding of antibody: antigen complexes [25]. This parity is thought to arise due to the presence of a mechanically strong clamp between the complementarity determining region in the antibody domains and the point of force application that prevents any force-induced remodelling of the complex. However, force effects can still be induced in antibody:antigen complexes by immobilising bivalent antibodies onto surfaces displaying epitopes at regular, but non-ideal, intervals. The strain introduced into immobilised antibodies decreases complex affinity to such an extent that, when imaged by fast scan AFM, IgGs display bipedal stochastic ‘walking’ [26]. The F_ab_ variant of the same IgG immobilised onto the same surface, by contrast, remained stationary. Force can thus act allosterically to alter complex affinity markedly. Recent work on the Ca^2+^-activated actin-binding protein, gelsolin [27] has demonstrated that the effects of force can also be quite subtle. For this protein, application of a force as low as 10 pN was found to increase the binding affinity of gelsolin for Ca^2+^ more than five-fold, suggesting that gelsolin may be activated at lower [Ca^2+^] than previously recognised when subjected to tensile forces. Introduction of strain into an enzyme (via tethering the N- and C-termini to a DNA spring) can also alter enzyme activity [28,29], opening the door to force-modulated catalysis. As the strength of covalent bonds is also reduced under force (see [30–32]), it is not surprising that chemo-mechanical effects have been observed for reactions of small molecules [32]. For example, Fernandez and colleagues have shown, using AFM force measurements, that the catalytic rate of thioredoxin is force sensitive [33] and that this AFM technique can be used to probe catalytic mechanisms [34].

## Larger remodelling events

**Figure 3:**
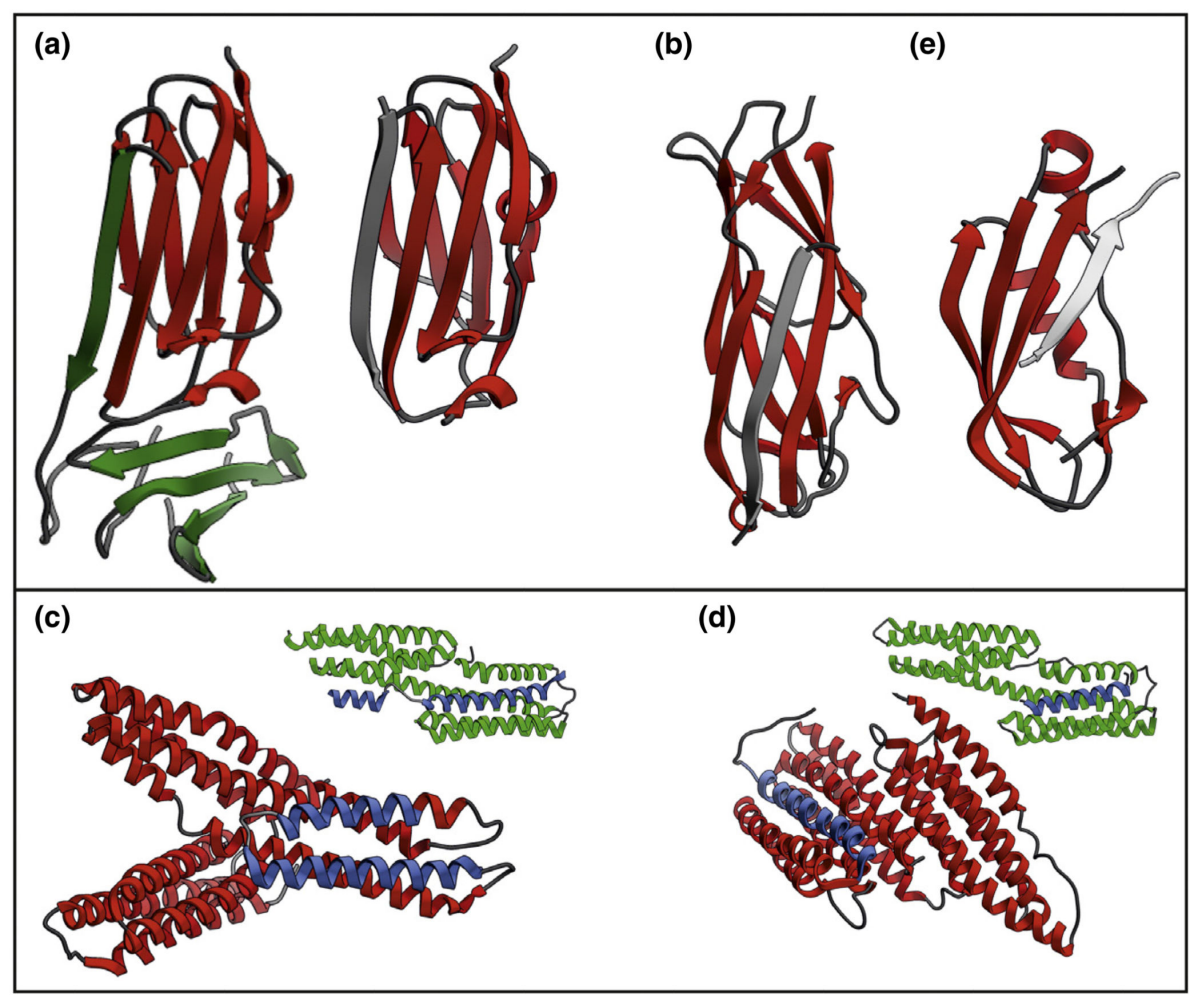
The structures of force-resistant and force-sensitive protein complexes. **(a)** in the absence of force, the N-terminal strand of FLNa20 (green, left) occludes the binding site for the integrin β-cytoplasmic tail (grey, right) on FLNa21 (red). **(b)** The bacterial Fim pilus is assembled by a donor strand complementation mechanism, whereby the Immunoglobulin-like fold of one domain (FimG in this case, red) missing the C-terminal β-strand is completed by the binding of an N-terminal extension of the subsequent Ig-like domain (FimF, grey). **(c)** and **(d)** upon mechanical extension, the cryptic vinculin binding sites (VBS, blue) within α-catenin ((c), red) and talin ((d), red) become accessible, triggering vinculin binding to the VBS (green and blue in the inset structures, respectively). **(e)** the complex formed between the C-terminal domain of TonB (red), tethered to the inner membrane of Gram negative bacteria and the TonB box (grey) of the outer membrane protein BtuB, that together span the periplasm. Structures drawn using UCSF Chimera [97] and PDB files:2J3S [98], 2BRQ [99], 2GSK [58], 3JWN [100], 4IGG [101], 4EHP [102], 1XWX (note that this is a theoretical model) [103] and 1 U6H [103].

At a longer length-scale force can induce conformational re-arrangements leading to allosteric activation or inhibition of a protein or protein complex. One notable case is titin which, in addition to its structural role, can act as a strain sensor, triggering muscle adaptation upon detection of mechanical strain. At physiologically relevant forces, low enough to maintain titin’s structural domains in the folded state (<50 pN), the C-terminal kinase domain unfolds via a multistep pathway. Early in the unfolding process the C-terminal tail of the kinase domain unravels from the remainder of the protein. Extension in this way activates the kinase by both allowing access of ATP to its binding site (which in the absence of force is occluded by the C-terminus) and by triggering autophosphorylation of a tyrosine residue that inhibits activity [35]. Force is also integral to outside-in and inside-out signal transduction between cells and their surroundings. Transmembrane proteins such as integrins are vital to this network, linking the extra-cellular matrix with the actin cytoskeleton. Many adaptor proteins are involved in this signal transduction pathway, with filamin playing a central role [36]. At the molecular level, filamin complex formation is driven by a β-strand augmentation of the 21st immunoglobulin-like domain of filamin A (FLNa21) by the β-integrin tail (Figure 3a). Under no force, FLNa21 cannot bind to integrin due to occlusion of the binding site by the N-terminal β-strand of the preceding filamin domain (Figure 3a). Rognoni *et al.* [37] investigated the mechanical behaviour of the auto-inhibited state and showed that the force-dependent gating characteristics of filamin allow for a cellular response to surprisingly low forces (the affinity for the C-terminal tail peptides of different interaction partners is increased up to seventeen fold upon increasing applied force from to 2 to 5 pN). The same authors then showed that switching between the auto-inhibited and activated state is enabled by cis–trans isomerisation of a proline residue in the force sensing domain (FLNa20), weakening the stability of the auto-inhibited state. Whilst cis–trans isomerisation does result in bond lengthening, the authors suggest that force induced unfolding accelerates this isomerisation, rather than force driving isomerisation *per se* [38^•^].

## Local unfolding prevents global unfolding or unbinding

In addition to direct signal transduction, mechanical deformation can also prolong bond lifetime (i.e. maintenance of native structure or complex) by reducing the level of force acting upon it. Such mechanisms have been postulated for the all-β proteins tensacin and Mel-CAM [39,40]. Given the relative strengths of α- and β-structures (see later) it may be expected that more sensitive safety latches could be achieved using the former, mechanically weaker, secondary structure. This is indeed the case for myomesin, a tandemly-arrayed dimeric multi-modular protein found in the M-band of the muscle sarcomere which has an unusual α-helical linker separating each Ig domain [41,42]. The α-helical segments were found to unfold at a much lower force than the Ig domains (24 and 83 pN, respectively [43]) and underwent folding/unfolding transitions at low loading rates. These helices, therefore, act as fast and reversible latches to ensure the structural integrity of the M-band.

Partial unfolding of one binding partner can also increase complex lifetime by decreasing the force being loaded onto it. For example, bacterial pili are long proteinaceous structures emanating from bacterial outer membranes that are used for a variety of functions including host colonisation. As this process may involve hydrodynamic shear forces, all of the non-covalent linkages connecting the host to the bacterium must be able to survive force-loading for effective colonisation. In type I pili, this is achieved by donor strand exchange between inherently mechanically strong Ig-like domains (Figure 3b) and the unwinding of the helical quaternary structure of the pilus at low force [44–46]. Type IV pili, involved in the pathogenicity of *Neisseria gonorrheae* and *Pseudomonas aeruginosa*, have a different quaternary structure, precluding such a mechanism. Instead, under force, these pili lengthen via a conformational re-arrangement and also display ‘nanospring’ behaviour (displacement proportional to force) with a spring constant of 2 pN nm^−1^ [47]. Similar (though indirect results) have recently been reported for the effects of inhibitor binding on the mechanical strength of domains 1 and 2 of CD4 (the primary receptor for gp120 on the surface HIV-1) [48]. These data suggest that HIV infectivity would be expected to increase with increasing length of the ‘tether’ connecting the virus to the cell, which has previously been observed [49].

## Can the unfolding behaviour of proteins be predicted?

As discussed above, different types of secondary structure behave differently under force. The relationship between structure and mechanical strength was studied in great detail soon after the emergence of force spectroscopy and a wide variety of protein structures have been unfolded by force methods (both experimentally and by simulations) since 1997 [50]. These studies have shown that α-helical proteins are mechanically weak, β-sheet proteins are mechanically strong, while proteins with mixed topologies display varied responses [51–54]. The difference in mechanical strength between proteins containing different secondary structural elements is thought to arise as a consequence of the localised nature of force application. In such a model, the height of the rate limiting transition state for unfolding is governed by the strength of interactions between amino-acids which bear the loaded force. The array of non-covalent interactions between adjacent β-strands in β-sheet proteins provides more stability against local mechanical deformation than the hydrophobic contacts between helices which can unfold in a stepwise, sequential manner. This difference appears to have been exploited by nature, as while both types of protein are used in mechano-transduction pathways, their roles are quite distinct. The low mechanical strength of α-helical proteins is utilised to facilitate unfolding, allowing exposure of novel binding sites for proteins that either strengthen these complexes or trigger a signalling event. For example, α-catenin (Figure 3c) and talin (Figure 3d) are all α-proteins which cross link cadherins to actomyosin or integrins to actin, respectively. Each, therefore has a key role in mechano-sensing (at cell–cell adhesions and focal adhesions, respectively). Using magnetic tweezers and AFM, talin was found to unfold at ~40 pN [16], whereas α-catenin unfolded in three steps: a reversible step at around 5 pN and two non-equilibrium steps at 10–15 pN [55^•^]. The outcome for both proteins is similar in that the initial unfolding events expose cryptic binding sites for vinculin (Figure 3c,d), a protein that stabilises adhesions, converting force to a biochemical signal. β-sheet proteins, by contrast, are employed if the non-covalent complex is required to resist breakage. As described for filamin above, many such interactions are mediated by β-strand augmentation or complementation [56], whereby a single β-stranded peptide binds to the β-sheet of its partner, forming a mechanically long-lived complex. Such interactions are observed for filamin:integrin, type I bacterial pili (Figure 3b) [57], and interactions between proteins that span the periplasm of Gram negative bacteria (Figure 3e) [58]. Our understanding of the mechanical stability of small, topologically simple domains has also led to the ability to design protein-based hydrogels, with dramatically improved flexibility and toughness [59^•^].

**Figure 4:**
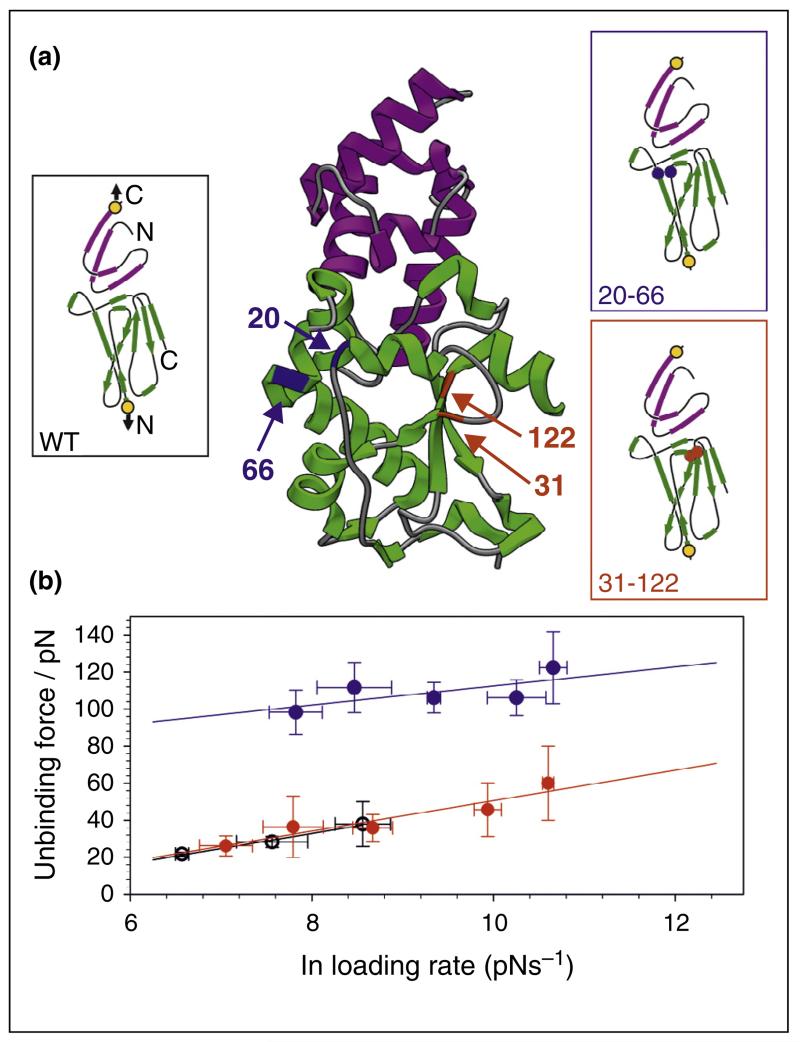
The highly avid E9:Im9 complex is a force-sensitive trip bond [73]. (**a**) When extended between residue 3 of E9 (green) and 81 of Im9 (pink), the complex dissociates at low force with a short lifetime (12.5 ms under 20 pN force) due to force-induced remodelling of the binding interface which is connected directly to the N-terminus of E9. (See left hand inset showing simplified topology diagram for the complex. Yellow filled circles designate pulling points). Interface remodelling can be prevented by introducing a disulfide cross-link (between residues 20 and 66, highlighted in blue and blue circles in the structure and topology diagrams, respectively), diverting the force propagation network away from regions proximal to the interface. This results in a mechanically strong, long lived complex (3.9 hours under 20 pN force), with a dissociation rate constant identical to that measured by ensemble methods. If a disulphide bond is introduced between residues 31 and 122 of E9 (red and red circles in the structure and topology diagrams, respectively), remodelling is not prevented, and the complex behaves identically to the wild-type (WT) protein. (**b**) Dynamic force spectrum of the wild-type complex (black circles), E9 cross-linked between residues 20–66 (blue closed circles) and 31–122 (red circles).

While protein topology governs the molecular response to extension to a large degree, the stability of the mechanical interface (the parts of a protein that resist the applied extension) can also affect protein mechanical strength and the degree of co-operativity upon unfolding. For example, molecular dynamics simulations demonstrated that protein L (a protein that is expressed on the outer cell wall of some bacteria but has no known mechanical function) unfolds by the shearing of two mechanical subdomains with an interface between neighbouring anti-parallel N- and C-terminal β-strands [60]. Increasing the hydrophobic contacts (or inter-digitation of side-chains) across this interface increased the mechanical strength of protein L from 134 to 206 pN [61]. *In vitro* unfolding studies on simple protein polymers have also shown that the mechanical strength of a protein depends on the direction of force application relative to the topology of the secondary structure. A protein may thus be able to resist mechanical deformation when force is applied in one geometry, but be weak when force is applied in another direction (similar to pulling apart Velcro). The anisotropic response of proteins and their complexes to force has now been demonstrated many times [62–73]. These effects, which to a large part were delineated using engineered model poly-proteins unfolded by AFM *in vitro*, have led to the realisation that proteins and their complexes may exploit different unfolding pathways in the presence and absence of force, leading to force-catalysed or force-triggered phenomena *in vivo* [36,73]. For example, we have shown that mechanical perturbation remodels the interface of an exceedingly stable complex (*K_d_* = 10^−14^ M, *k*_off_ = 1.8 × 10^−6^ s^−1^) formed between the bacterial antibiotic nuclease colicin E9 and its inhibitor, immunity protein 9 (Im9) so that dissociation occurs at a surprisingly low force (<20 pN) [73]. Examination of the N-terminal sequence of E9 (through which force or remodelling is applied or carried out *in vivo*) showed that this region docks against the remainder of the globular domain with little side-chain inter-digitation (see Figure 4). As described for protein L, this is ideal for transmitting mechanical signals to the binding interface at low force. Remodelling increases the off-rate a million-fold relative to that expected for a slip bond, allowing Im9 release and E9 activation at a biologically relevant rate upon binding to a competing organism.

## The effects of force on proteins are varied

As the properties of proteins and their complexes can differ in the absence and presence of mechanical deformation, these effects must be accounted for when force is thought to be present in a cellular context. This is illustrated in the investigation of adhesion junction assembly. *In vivo* experiments have shown that intercellular adhesion junctions are linked to the actin cytoskeleton via an E-cadherin:β-catenin:αEcatenin complex. This allows force transduction across cells, giving shape and driving morphogenetic changes during development. By contrast with *in vivo* experiments, this complex was found to bind to actin too weakly to allow mechano-transduction *in vitro*. As this complex presumably forms under tension *in vivo*, Buckley *et al.* [74^••^] investigated the effect of force by sequentially forming and breaking the interaction between E-cadherin:β-catenin:αEcatenin and an actin filament suspended between two optically trapped beads. Instead of a decrease in lifetime, as would be expected from a slip bond, the lifetime of the E-cadherin:β-catenin:αEcatenin:actin complex was found to increase under increasing force before decreasing like a slip bond. This type of interaction, called a ‘catch-bond’ [75], is reminiscent of a molecular-scale finger trap toy. Catch bonds are observed in several protein complexes that have evolved to withstand hydrodynamic shear forces such as the interactions that mediate the immobilisation of uropathogenic *E. coli* to bladder epithelial cells [76]) and leukocyte rolling on the extracellular surface of endothelial cells of blood vessels [77,78]. At the macroscopic level, a widely-accepted explanation of this phenomenon is that, under force, the proteins undergo topological rearrangements, transitioning from low to high affinity states [74^••^]. Manibog *et al.* [79] investigated Ca^2+^-dependent catch-bond formation between pairs of cadherins using both molecular dynamics simulations and AFM force spectroscopy. These investigations revealed that cadherins exhibit decreased conformational flexibility in the presence Ca^2+^. Application of force pulled these rigidified dimers into register, forming long lived, *de novo* hydrogen bonds. The same underlying mechanism (the *de novo* formation of force-induced interactions) was also recently proposed by applying a theoretical approach to analyse ligand–receptor protein complexes (selectin and integrin receptors) [80].

In addition to slip bonds and catch bonds, other more complex behaviour can be observed. Springer and colleagues [81] used laser tweezers to study the forced unbinding behaviour of the A1 domain of von Willibrand Factor from glycoprotein 1b α subunit (GPIbα) present on the surface of platelets. Two dissociation pathways were observed and while both behaved as slip bonds, each predominated at different force loading rates, with the more force resistant pathway being followed at higher loading rates. The authors termed such behaviour as a ‘flex bond’ and suggested that the second state may take part in the early events in platelet interactions. As described above, under mechanical extension of the N-terminus, the high affinity E9:Im9 complex dissociates at a low force (short lifetime) due to remodelling of the binding interface [73]. If this remodelling is prevented by locking the N-terminus to the rest of protein (via a disulfide bond between residues 20 and 66, Figure 4), the force required to unbind the complex increases from 34 to 102 pN at a force loading rate of 2980 pN s^−1^, yielding an off rate extrapolated to zero force that is identical to that measured by ensemble methods. These data suggest that the E9:Im9 complex demonstrates behaviour akin to a trip wire: in solution E9:Im9 is stable with a dissociation rate of 10^−6^ s^−1^ (which can only be measured in AFM experiments by introducing specific cross-linking). Upon binding to a competing bacterium, remodelling of the E9:Im9 interface converts a complex that is very stable in the absence of force to a labile one, leading to colicin activation and cell death. We term such interactions ‘trip bonds’. For colicin function, a trip bond meets the seemingly mutually exclusive requirements of providing long term protection to the host, yet permiting the facile dissociation of immunity protein that is required for cell invasion of bacterial competitors.

## Membrane proteins as mechanosensors

As expected from their location at the cell boundary, membrane proteins are also used as force sensors for both signal transduction and homeostasis. In principle, a mechanical signal may drive conformational changes by altering the lipid bilayer (by changes in curvature stress, bilayer thinning and lipid composition [82]), by direct mechanical activation, by gating of intra- or extra-cellular domains, or by a mixture of all three. Gating is exemplified by ankyrin repeat sequences found in cytoplasmic domains of transient receptor potential (TRP) channels. These proteins were found, using AFM [83], to behave as nano-scale Hookean springs and are thus candidates for gating tethers for mechanoreceptors in sensory hair cells as well as in *Drosophila* bristles [84–86]. The yeast transmembrane Wsc1 cell surface sensor displays similar nano-spring behaviour whose stiffness is sensitive to growth conditions [87].

While single molecule force experiments have been used to investigate the extension of soluble nano-springs and the mechanical stability of membrane proteins [88,89], other techniques are required to test the ‘force-from-lipid’ principle [90]. Patch clamp methods can measure the effect of lipid composition and tension (by changing the pressure applied to the patch) on the activity or one or more channels. In many ways patch clamping can be regarded as the first single molecule technique and was used in the early 1990s to show that the bacterial membrane channel protein, MscL, switched between closed and open states upon application of tension when reconstituted into synthetic liposomes [91]. Later work has shown, as expected, that liposome composition affects the electrophysiological properties of this protein and MscS, another mechanosensitive bacterial membrane protein [92]. By sensing membrane tension, MscL acts as a safety valve, allowing passage of solutes into bacteria when they encounter a hypo-osmolar environment. The activity of TRAAK and TREK1 K^+^ channels has also been reported to be modulated by membrane tension [93,94] and composition [94], demonstrating that eukaryotic organisms also utilise these force sensors to modulate or control biological function.

## Conclusions and perspectives

The ability to manipulate single proteins and their complexes has led to an understanding of the effects of force on ‘bond’ lifetime. These studies, performed *in vitro* on either simple model proteins or minimal ‘*in vivo*’ models, have revealed a rich response of proteins and their complexes to mechanical deformation. These effects are diverse in nature and can be large in magnitude. Thus it is vital to integrate force data with those derived from traditional ensemble methods to fully understand systems in a cellular context. To do this, the force applied to proteins *in vivo* must be quantified for the system under study. Currently it remains challenging to use standard force probes *in vivo*. Progress, however, is being made in quantifying force *in vivo*. Force spectrum microscopy, has revealed that the activity of cellular motors is the dominant cause of force fluctuations *in vivo* and that the magnitude of the fluctuation can be related to the physiological status of the cell [95]. Direct readout of applied forces *in vivo* has been achieved using two related, but distinct, fluorescence techniques that utilise the ability of Förster resonance energy transfer methods to report on changes in distance induced in a biosensor of known mechanical strength. Gratifyingly, these studies have shown that the forces applied in extracellular adhesion, in Notch signalling and across vinculin in focal adhesions are similar to those being measured *in vitro* (~12, 40 and 2.5 pN, respectively) [23,96]. The ability to calibrate the force-induced effects observed for a particular system *in vitro* to the precise force levels applied to the same system *in vivo* is very powerful and will yield insight into the rich and varied effects of force in nature.
